# Two-Dimensional Metals Over, Inside, or Beneath Templates

**DOI:** 10.34133/research.0790

**Published:** 2025-08-05

**Authors:** Jinbo Pang, Shuye Zhang, Yufeng Hao, Hong Liu, Mark H. Rummeli, Weijia Zhou, Rafael G. Mendes

**Affiliations:** ^1^ Institute for Advanced Interdisciplinary Research (iAIR), University of Jinan, Jinan 250022, China.; ^2^ State Key Laboratory of Precision Welding and Joining of Materials and Structures, Harbin Institute of Technology, Harbin 150001, China.; ^3^ Chongqing Research Institute of HIT, Chongqing 401135, China.; ^4^ National Laboratory of Solid State Microstructures, College of Engineering and Applied Sciences, Jiangsu Key Laboratory of Artificial Functional Materials and Collaborative Innovation Center of Advanced Microstructures, Nanjing University, Nanjing 210023, China.; ^5^ State Key Laboratory of Crystal Materials, Shandong University, Jinan 250100, China.; ^6^ Soochow Institute for Energy and Materials Innovation, College of Energy, Key Laboratory of Advanced Carbon Materials and Wearable Energy Technologies of Jiangsu Province, Key Laboratory of Core Technology of High Specific Energy Battery and Key Materials for Petroleum and Chemical Industry, Soochow University, Suzhou 215006, China.; ^7^ Electron Beam Emergent Additive Manufacturing (EBEAM) Centre, Institute of Environmental Technology (IET), Centre for Energy and Environmental Technologies (CEET), VSB—Technical University of Ostrava, 708 33 Ostrava, Czech Republic.; ^8^ Institute for Materials Chemistry, Leibniz Institute for Solid State and Materials Research Dresden (IFW Dresden), Dresden 01069, Germany.; ^9^ Interfaces, Confinement, Matériaux et Nanostructures, CNRS-Orléans, UMR7374, Orléans 45071, France.

## Abstract

Two-dimensional (2D) metals have drawn great attention because of their extraordinary properties, especially in applications that favor van der Waals interaction. The development of advanced characterization tools has facilitated the understanding of formation or growth mechanisms of 2D metals. In this perspective, we discuss 5 common approaches to obtaining 2D metals, including, (top down) van der Waals squeezing and selective extraction, and (bottom up) electron beam-induced growth, self-assembly, and graphene-templated wet chemistry growth. The future opportunities are proposed in the summary section. Furthermore, challenges and problems such as thermodynamic stability and scalability in 2D material growth are proposed for the community to tackle.

## Background

Two-dimensional (2D) materials, e.g., graphene, are first predicted to be unstable, viz., thermal fluctuations of their lattice lead to the collapse of the structure. Then, the cleavage of graphite by using Scotch tape has succeeded in isolating monolayer graphene.

Unlike graphene, metals are often close packed due to nondirectional metallic bonds. Therefore, 2D metals cannot be exfoliated from 3D bulk metals without layered structure. The terminology “2D metals” refers to the one-atom-thick elemental metals [1], which is analogous to graphene, one-atom-thick carbon nanosheet of hexagonal lattice. 2D metals are a geometry concept of nonlayered structure. Previously, the concept of Xenes is proposed [2] for summarizing the monoelemental 2D materials, including both nonmetals, e.g., B (borophene), C (graphene), Si (silicene), Ge (germanene), P (phosphorene), and As (arsenene), as well as 2D metals, e.g., Sb (antimonene) and Sn (stanene). Recently, plumbene (Pb) is experimentally synthesized by segregation mechanism with theoretically calculated bandgap. Here, we summarize the category of one-atom-thick metals as 2D metals. Sometimes, one may expand the 2D metal concept to thickness of a few atoms (e.g., nanosheets), similar to few-layer graphene (<10 layers).

Interestingly, 2D metals shows superior potentials in applications that favor large specific area. Indeed, bulk 3D metals often play the role of conducting electrodes in electronics and electrochemistry. When thinning the thickness, ultrathin 2D metal films provide a new physical platform for investigating emerging phenomenon [3], such as surface and edge conductivity, quantum optics, large band gap (6 eV, dielectrics) [4], and plasmonics. Besides, large specific area of 2D metals facilitates the surface reaction in electrochemistry for energy conversion including catalysis and fuel cells.

Ultrathin metal films (down to a few nanometers thick) can be fabricated by conventional thermal deposition methods. Thin solid films, including metals, can be grown by conventional deposition methods, such as sputtering, electron-beam evaporation, and physical vapor deposition. Often, the metal films are micrometer thick with nanocrystalline or microcrystalline feature, which serves as electrodes for electronic devices. By pulsed physical vapor deposition, ultrathin Te nanowires (quasi-metal metalloid) can be synthesized with a 10-nm thickness [5]. When decreasing the thickness of metals to 4 nm, incomplete films form with discrete islands [6], analogous to dewetting behavior, which could be attributed to the surface/interface stress from change in surface energy during the continuous metal deposition.

Also, ultrathin metal films can be fabricated by atomic layer deposition, e.g., Ru (3 nm) [7], Ir (1 nm), and Pt (2.5 nm) [8]. The deposition mechanism is the chemisorption of metal–organic coordination complex over substrates and thermal decomposition of the organic ligands from central metal atoms.

By molecular beam epitaxy, Jia and colleagues [4] fabricated 2D Sn monolayer (stanene) over Bi_2_Te_3_ (111) substrates. The biatomic-layer structure of stanene shows height difference (0.12 nm) between these 2 atomic layers of Sn. The deposition rate of Sn metal minimizes at 0.4 monolayer min^−1^, as evaporated from effusion cell. Scanning tunneling microscope (STM) images of 2D Sn/Bi_2_Te_3_ species exhibit the alignment of Sn atoms, vertically located above each Te atom (the top layer of Bi_2_Te_3_), probably due to the little lattice mismatch between these 2 crystals [4].

Many 2D metals suffer from oxidation and degradation upon exposure in ambient conditions [9]. In early stage, 2D metals are characterized by STM tool in ultrahigh vacuum condition. Indeed, one-atom-thick Pb and In films are fabricated over Si (111) surface by molecular beam epitaxy method [10]. For device fabrication, one may overcome the oxidation challenges by encapsulating 2D metals with inert materials, e.g., h-BN or graphene. Thus, the h-BN encapsulated 2D metal Bi (5 nm) was fabricated by van der Waals molding [11]. Besides, confined heteroepitaxy occurs for atomically thin 2D metal growth at the graphene/SiC interfaces [12,13].

In this perspective, we briefly introduce 5 methods for fabricating 2D metals. Two paths exist for nanomaterials fabrication, i.e., top-down and bottom-up. The van der Waals squeezing and selective extraction can be classified into top-down methods. Then, electron beam-induced 2D metal growth, graphene-templated Au nanosheets, self-assembly, and CO reduction of Pd nanosheets can be categorized as bottom-up methods.

Figure shows the 5 methods for obtaining 2D metals. They are electron beam irradiation-induced 2D metal membrane over graphene pores [14], extrusion of liquid metal between 2 MoS_2_ anvil [15], graphene-templated Au nanosheet [16], carbon monoxide-reduced Pd nanosheets [17], and selective extraction of Au monolayer from Ti_3_AuC_2_ MXene sheets [18] (from top to bottom). Now, we briefly introduce the different methods for preparing 2D metals.

**Figure.**
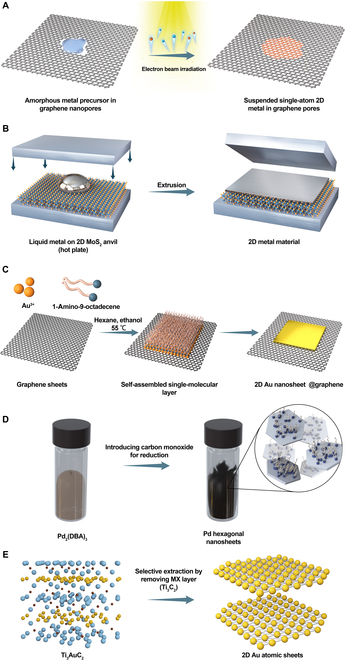
Common strategies for obtaining 2D metals. 2D metal membrane (A) inside graphene pores, (B) beneath anvil, (C) over templates, (D) self-assembly, and (E) selective extraction.

First, we come to discuss electron beam-induced 2D metal membranes inside graphene pores.

## Graphene Nanopore-Confined 2D Metals

On chemical vapor deposition (CVD)-grown graphene samples, one can often observe Fe species by element analysis in a transmission electron microscope (TEM). Upon electron beam shower, Zhao et al. [14] observed a freestanding 2D iron membrane confined in a graphene nanopore within TEM. Here, the 2D Fe membrane was transformed from Fe adsorbents introduced during graphene transfer onto TEM grids, i.e., etching Cu foil substrate with FeCl_3_ etchant. Other 2D metals can be analogously produced such as Cr, Mo, Au, Zr, and even 2D compounds, including Mo_2_C and ZnO [19–23]. Such a confinement approach may have stemmed from the injection of metal atoms or fullerenes inside carbon nanotubes like peapods [24]. Also, one can imagine from materials science theory that condensation of nanoparticles occurs upon sintering in a confined space. In contrast, the outer surface of carbon nanotubes has served as a template for producing MoS_2_ or h-BN nanotubes [25].

## 2D Metals Squeezed by Sapphire/MoS_2_ Anvils

Recently, Zhang and colleagues [15] proposed a squeezing strategy for producing 2D metals in the interfaces of 2 MoS_2_ nanosheets. Here, metal pieces on a hot plate are melted into liquid and squeezed into a thin planar membrane by 2 MoS_2_-coated sapphire substrates, termed van der Waals anvils. Due to the coating of MoS_2_ epitaxially grown over a sapphire surface, one can achieve the atomically smooth surface of 2D metals by high-pressure squeezing. Here, the liquid metal often refers to Ga with a melting point around room temperature (30 °C). Other metals also show success in 2D metals [15] upon high-pressure squeezing, e.g., In, Sn, Bi, and Pb, with their melting point, 157, 232, 272, and 327 °C, respectively.

The idea may originate from the low-resistance metal/semiconductor contact in fabricating transistor devices. Indeed, metal and semiconductor contact in 2D material-based transistors become vital for elevating device performances. Such 2D metals may substitute the conventional low-melting-point metal alloys in electronic packaging as solder materials for electrical interconnect [26–28].

## Other Approaches for Obtaining 2D Metals

### Selective extraction

Selective extraction often refers to the extraction of A layer from MAX phases by removing M and X elements, e.g., extracting Au layer from Ti_3_AuC_2_ phase. Here, Ti_3_AuC_2_ was synthesized by ion exchange intercalation of Ti_3_SiC_2_ MAX phase (the fifth row of Figure). Second, the Ti and C layers are selectively removed from Ti_3_AuC_2_ in etchants, such as Murakami’s reagent and cetrimonium bromide [18]. Eventually, 2D Au (goldene) forms and evidenced in the cross-section TEM images. This approach minimizes the consumption of Au in electrode applications of electronic devices.

Starting from the MXene monolayer Mo_2_Ti_2_C_3_, one can obtain nanoribbons of individual elements, e.g., graphene of C element or Mo nanoribbon. Here, Mo nanoribbon can be classified as 2D metal [29]. After irradiation of the electron beam for 2 min, the species experience structural transition, and atomic reconfiguration occurs. Eventually, Mo nanoribbon forms inside MXene nanopores.

Another example of selective extraction is Ag nanosheets by selective etching of Al in repeated folding and calendaring thinned Ag–Al stacked layers [30].

### Growth over graphene templates

Metal Au possesses a face-centered cubic (fcc) phase. The Au square nanosheets were grown on templates [16]. First, Au^3+^ from HAuCl_4_ converts to Au^+^, which coordinates with surfactant into an AuCl–ligand complex on graphene. Then, the Au nanosheets (2.4 nm thick, 200 nm large) can be synthesized after removing the surfactant.

### Self-assembly monolayer of metal precursors and CO reduction

The Pd complexes can chemisorb at the surface of cetyltrimethylammonium bromide (CTAB; a surfactant), which experiences ligand exchange and reduction by CO introduction [17]. Eventually, Pd hexagonal nanosheets form from the emulsions proposed by Remita and colleagues [17].

## Emerging Trends

Exciting approaches to synthesize 2D metals are summarized in this perspective. Also, the representative metal types of each method are listed. Besides, 2D metals show great applications in catalysis, superconductivity, and electric interconnect. Indeed, 2D metals are ideal contact electrodes for electronic and optoelectronic devices with 2D semiconductor channel materials. The van der Waals interaction may facilitate the low-resistance ohmic contact by suppressing the Schottky barrier [31].

However, 2D metals research still remains in its infancy stage. Thus far, a few one or few-atom-thick 2D metals are experimentally synthesized, including Bi, Pb, Sb, Sn, In, Ga, Fe, Pt, Pd, Au, Ag, Ir, and Ru. Theoretical calculation shows mechanical stability of 45 kinds of 2D metal patches suspended over graphene pores [32]. These mechanically robust 2D metals may also expand into large area film by van der Waals squeezing at liquefaction temperatures. In terms of growth, many hurdles require efforts to overcome, including thermodynamic stability, thickness regulation, monolayer growth, and upscale production. For conventional thermal deposition approaches, the suppression of surface/interface stress by modulating metal/substrate interaction may improve the quality of metal film with single or bi-atomic thickness. By molecular beam epitaxy method, more 2D metals can be deposited in templated growth fashion over other smooth and flat surfaces of single crystals, such as graphene, InSb, PbTe, and Ag.

For the applications, their intrinsic material properties should be regulated for engineering interfaces and integrating into devices. For example, the catalytic capability of 2D metals may be quantified and standardized compared to conventional nanoparticles and clusters [33]. Besides, artificial intelligence for computing may assist the discovery of new types of 2D metals [34]. Also, future efforts may be put into exploring the electronic, photonic, catalytic, and superconductivity performances of 2D metals.
